# Limb-Sparing Reconstruction for Chronic Non-Bacterial Osteomyelitis of the Toe in a Pediatric Athlete: A Case Report

**DOI:** 10.3390/reports9010032

**Published:** 2026-01-23

**Authors:** Alan E. Augdahl, Thuy-Mi Le, Aamir Ahmed, Rahul Mittal

**Affiliations:** 1 Department of Surgery, Podiatric Medicine, Cooper Barnabas Medical Center, Livingston, NJ 07039, USA; 2 Department of Surgery, Robert Wood Johnson School of Medicine, Piscataway, NJ 08854, USA; 3 Department of Health Informatics, School of Health Professions, Rutgers University, Piscataway, NJ 08854, USA

**Keywords:** chronic non-bacterial osteomyelitis, digital fusion, calcaneal bone grafting, case report

## Abstract

**Background and Clinical Significance:** Chronic non-bacterial osteomyelitis (CNO) is a rare autoinflammatory bone disorder that primarily affects children and adolescents, with females more frequently impacted. The condition remains poorly understood, though cytokine dysregulation and inflammasome activation are believed to contribute to its pathogenesis. Clinically, CNO is often difficult to distinguish from infectious osteomyelitis, as presenting symptoms such as bone pain, swelling, and functional limitation are nonspecific, while cultures are frequently negative. As a diagnosis of exclusion, delays in recognition can lead to prolonged or unnecessary antibiotic exposure and uncertainty in management. **Case Presentation:** A 14-year-old male with a history of left second toe osteomyelitis initially diagnosed in 2021. Despite negative cultures and limited histopathologic findings, he received multiple antibiotic courses with little improvement, and the digit remained chronically swollen. Three years later, a repeat evaluation revealed osseous resorption of the middle and distal phalanges, and a biopsy confirmed acute and mild chronic fibrosing osteomyelitis, consistent with CNO. Given the risk of progression and possible amputation, surgical reconstruction was pursued. The patient underwent autologous calcaneal bone grafting with digital fusion using a K-wire. At three months and one year postoperatively, radiographs demonstrated solid fusion of the digit with maintained activity and resolution of pain. **Conclusions:** This case emphasizes the diagnostic complexity of CNO and the importance of considering it in children with culture-negative or recurrent osteomyelitis. It further illustrates how timely surgical intervention can preserve function and quality of life while avoiding unnecessary amputation.

## 1. Introduction and Clinical Significance

Chronic non-bacterial osteomyelitis (CNO) is a chronic, sterile, autoinflammatory bone disorder primarily affecting children and adolescents, most often females [1,2]. The disease exists on a clinical spectrum, with chronic recurrent multifocal osteomyelitis (CRMO) representing the most severe form [3]. Although once considered rare, CNO is now increasingly recognized due to improved clinical awareness and increased use of whole-body magnetic resonance imaging (MRI) [4]. The underlying pathophysiology remains incompletely understood; however, current evidence suggests that dysregulation of the innate immune system and inflammasome activation are involved, indicated by increased IL-1β and TNF-α activity, as well as reduced anti-inflammatory cytokines such as IL-10 and IL-19 [5,6]. Genetic variants within LPIN2, IL1RN, and FBLIM1 have further linked CNO to the broader category of monogenic autoinflammatory syndromes [7]. Clinically, CNO presents with insidious bone pain and localized swelling. Systemic symptoms are generally mild. Radiographs typically demonstrate mixed lytic and sclerotic lesions with periosteal reaction, while histopathology shows chronic inflammation without bacterial growth. These findings are nonspecific, and CNO is frequently misclassified as infectious osteomyelitis, resulting in recurrent diagnostic delays and unnecessary courses of antibiotics [8,9].

Historically managed with prolonged antibiotics that have had limited effect, current treatment now emphasizes immunomodulatory strategies rather than antimicrobial therapy. First-line management typically involves nonsteroidal anti-inflammatory drugs (NSAIDs), with corticosteroids, disease-modifying antirheumatic drugs (DMARDs), bisphosphonates, or biologic agents reserved for refractory disease [10,11,12]. Although most cases are treated medically, surgery may be required for biopsy, drainage of sterile abscesses, or reconstruction when bone integrity is compromised [13]. Autologous calcaneal bone grafting provides a practical option for reconstructing pediatric forefoot defects due to its proximity, low morbidity, and reliable osteogenic properties [14]. Limb-sparing reconstruction for isolated phalangeal destruction in pediatric CNO has rarely been reported. To our knowledge, there is no literature reporting digital reconstruction using autologous calcaneal bone graft in pediatric CNO, and this may represent a valuable limb-preserving option in selected patients with progressive osseous destruction.

## 2. Case Presentation

A 14 y.o. male with a history of left second toe osteomyelitis in 2021 presented to the emergency department (ED) in 2024 with concerns for osteomyelitis of the same left second toe. He was previously admitted in 2021 for complaints of left foot pain and left second toe swelling. The pain started after playing basketball, making it difficult for him to bear weight. Upon arriving home, he noticed that his left second toe had become swollen, and he took Advil with some relief. His pain returned the following morning, leading him to present to the ED. At this time, he reported 6 out of 10 pain when trying to bear weight on his foot. Denied any fever, numbness, or tingling. On imaging, “X-rays of the left foot showed no fractures but were concerning for osteomyelitis.” Erythrocyte sedimentation rate (ESR) 16 mm/h and C-reactive protein (CRP) < 0.29 mg/L. Cultures from both blood and tissue yielded no growth. Bone biopsy of the toe demonstrated “remodeling changes and few scattered neutrophils” without evidence of acute infection. The patient received a 5-day course of ceftriaxone, a 4-day course of vancomycin, and a 1-day course of cefazolin as an inpatient and then switched to an 8-day outpatient course of cephalexin. According to the caregiver’s report, antibiotic exposure lasted approximately 1 month, despite persistently negative cultures. The patient was then followed by an orthopedist for 1 year and was told that the toe would continue to be large and swollen for the remainder of his life.

**Figure 1:**
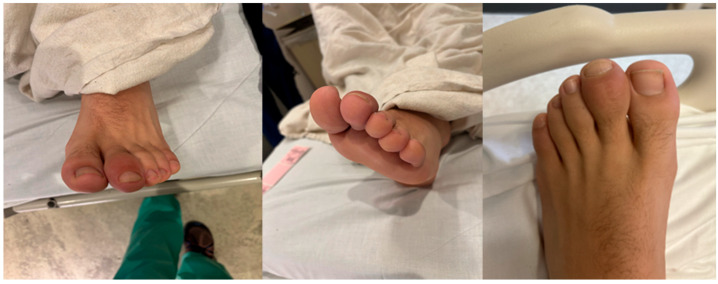
Initial clinical presentation showing swelling and enlargement of the left second toe in 2024.

Due to continued enlargement and functional concerns, the family sought further evaluation by a podiatrist in 2024. Upon presentation, the patient denied experiencing fever or pain and reported ongoing participation in sports, including running and soccer (Figure 1).

**Figure 2:**
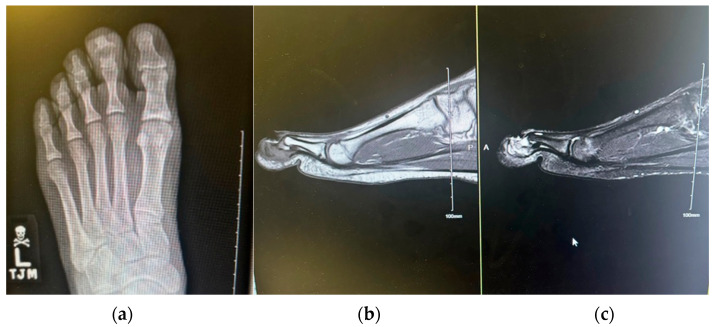
Initial imaging demonstrating osseous abnormalities of the left second toe. (**a**) Plain radiograph showing osseous resorption of the middle and distal phalanges with associated soft tissue swelling (**b**) Sagittal T1-weighted magnetic resonance image demonstrating hypointense marrow signal within the middle and distal phalanges. (**c**) Sagittal T2-weighted magnetic resonance image demonstrating corresponding hyperintense signal consistent with an inflammatory osteomyelitic process. The proximal phalanx and metatarsal demonstrate preserved marrow signal for comparison.

An X-ray of the toe was ordered, which read as “Osseous resorption of the left second middle and distal phalanges along the widened distal interphalangeal joint with surrounding soft tissue swelling. In the proper clinical setting, findings may be related to underlying infectious process/septic arthritis” (Figure 2).

**Figure 3:**
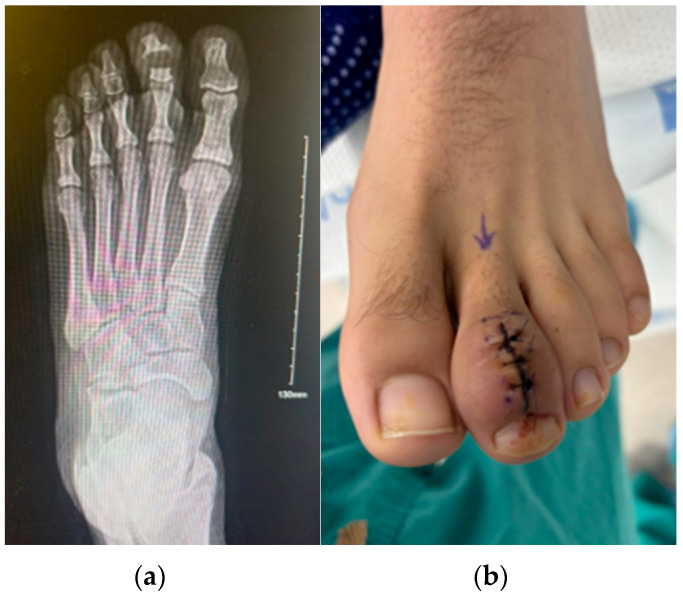
(**a**) Post-bone biopsy radiographs. (**b**) Clinical photograph demonstrating persistent swelling and structural changes.

Following the advice of podiatry, he presented to the ED, where he denied any fever or pain. ESR 11 mm/h, CRP 0.29 mg/L, and white count 7.3 × 10^9^/L. A bone biopsy revealed acute and mild chronic fibrosing osteomyelitis, supporting a diagnosis of CNO by exclusion (Figure 3).

No standardized quantitative measurements (e.g., caliper-based circumference or volumetric assessment) were obtained to objectively quantify the degree of digital inflammation, representing a limitation of this report.

Given that conservative treatment with nonsteroidal anti-inflammatory drugs over a three-year period without sustained clinical improvement had not been effective, the decision was made to proceed with surgical intervention per the patient’s and family’s wishes. Although pediatric rheumatology consultation can be valuable in the multidisciplinary care of CNO, further referral in this case was felt unlikely to alter management, given the patient’s three-year history of symptoms, failure of prolonged NSAID therapy, and progressive radiographic changes, and it could have resulted in unnecessary delay in definitive treatment. The patient did not receive bisphosphonate or biologic therapy prior to surgery. This decision was based on the localized nature of the disease confined to a single digit, the prolonged three-year disease course with progressive structural bone loss, and the absence of evidence demonstrating that immunomodulatory therapy can reverse advanced focal osseous destruction of small phalanges. While bisphosphonates and biologics have demonstrated efficacy in multifocal or systemic CNO, reports addressing their use in isolated digital involvement are rare and primarily describe symptomatic improvement and reduction in inflammatory MRI signal rather than restoration of structural bone integrity [15].

A limb-sparing reconstructive approach was pursued, given the progressive phalangeal osseous loss and risk of potential future amputation. The previous surgical incision over the left second digit was reopened, and the digit was systematically evaluated for tissue viability and evidence of active infection. Upon entry, a coagulated hematoma was identified and removed. The subcutaneous tissues and deep fascia were exposed using sharp and blunt dissection, with careful preservation of neurovascular structures. The digit was elevated and exsanguinated with an Esmarch bandage, and a thigh tourniquet was inflated to 300 mmHg to maintain a bloodless field.

Intraoperatively, hypergranular tissue was present at the site of prior bone resection, but there was no purulence, abscess formation, or necrotic bone, further supporting a non-infectious inflammatory etiology. The extensor digitorum longus tendon was identified and mobilized to allow direct visualization of the resected distal interphalangeal joint. The previous graft was removed, and the bone ends were curetted to healthy, bleeding bone. The joint space was measured and irrigated with copious sterile solution.

**Figure 4:**
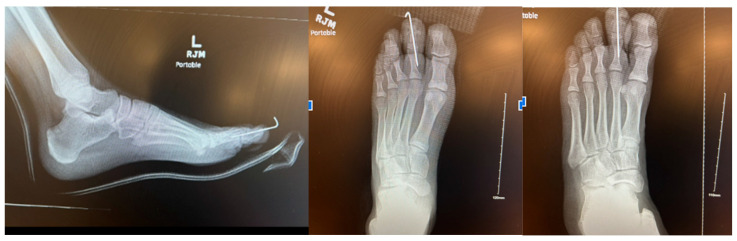
Intraoperative photographs showing graft harvest, defect preparation, and K-wire arthrodesis for digital reconstruction.

A separate incision was made over the posterolateral calcaneus, and a corticocancellous bone graft was harvested using an osteotome and mallet, with care to avoid neurovascular injury. The donor site was filled with a demineralized bone matrix and irrigated. Vancomycin powder was applied to both the donor and recipient sites to minimize the risk of postoperative infection. The bone graft was shaped and placed into the resected joint space. A 0.062-inch (1.6 mm) K-wire was then advanced in a retrograde fashion through the graft and into the middle phalanx, providing stable internal fixation (Figure 4).

**Figure 5:**
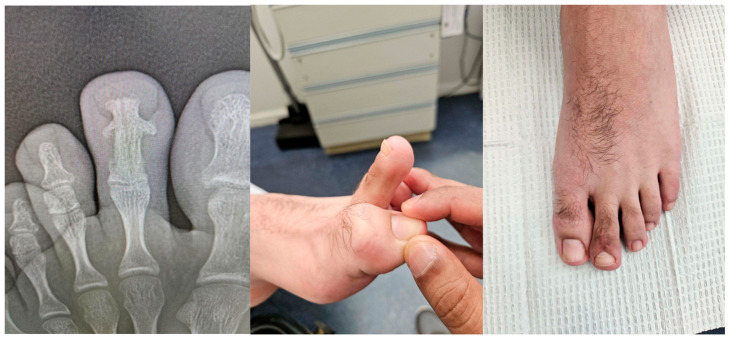
Three-month postoperative radiograph demonstrating solid fusion at the distal interphalangeal joint.

The digit was aligned in a rectus position, and fixation was confirmed through intraoperative imaging. The surgical site was closed in anatomic layers with absorbable and non-absorbable sutures under minimal tension. Intraoperative cultures and tissue samples were obtained for microbiological and histopathological analysis. The patient remained non-weight-bearing for four weeks, followed by a gradual return to activity. X-rays 3 months after the operation revealed the digit to be fused (Figure 5).

**Figure 6:**
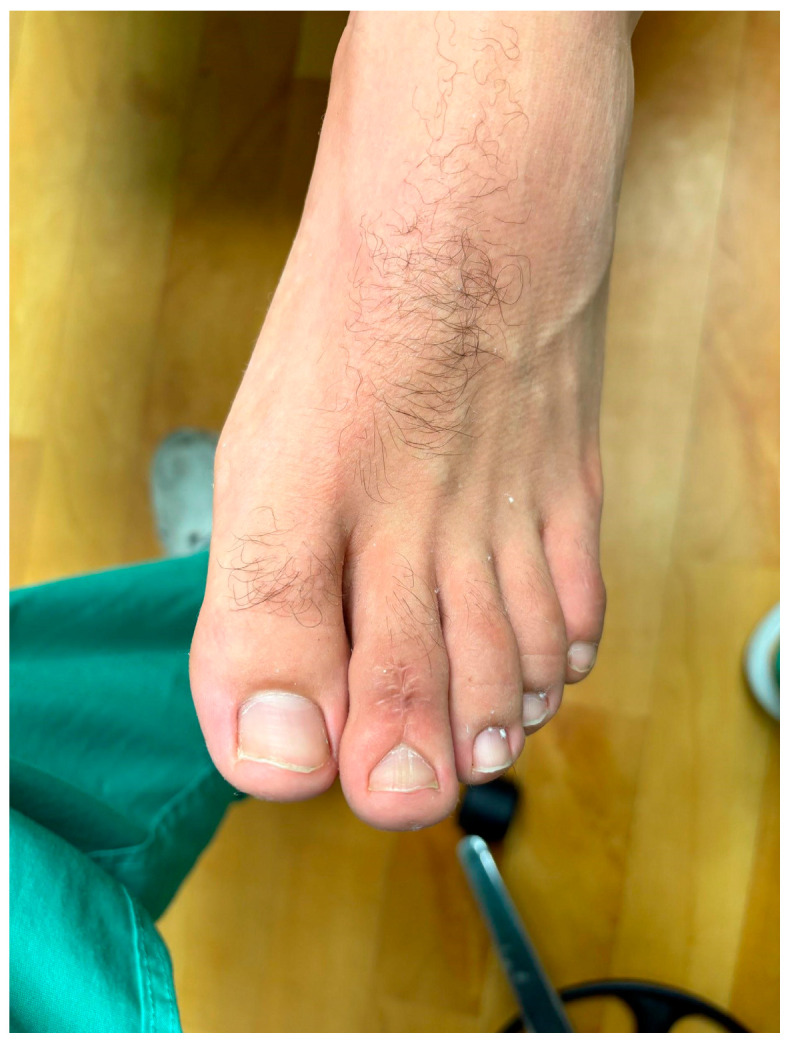
One-year clinical photograph showing restored toe profile.

**Figure 7:**
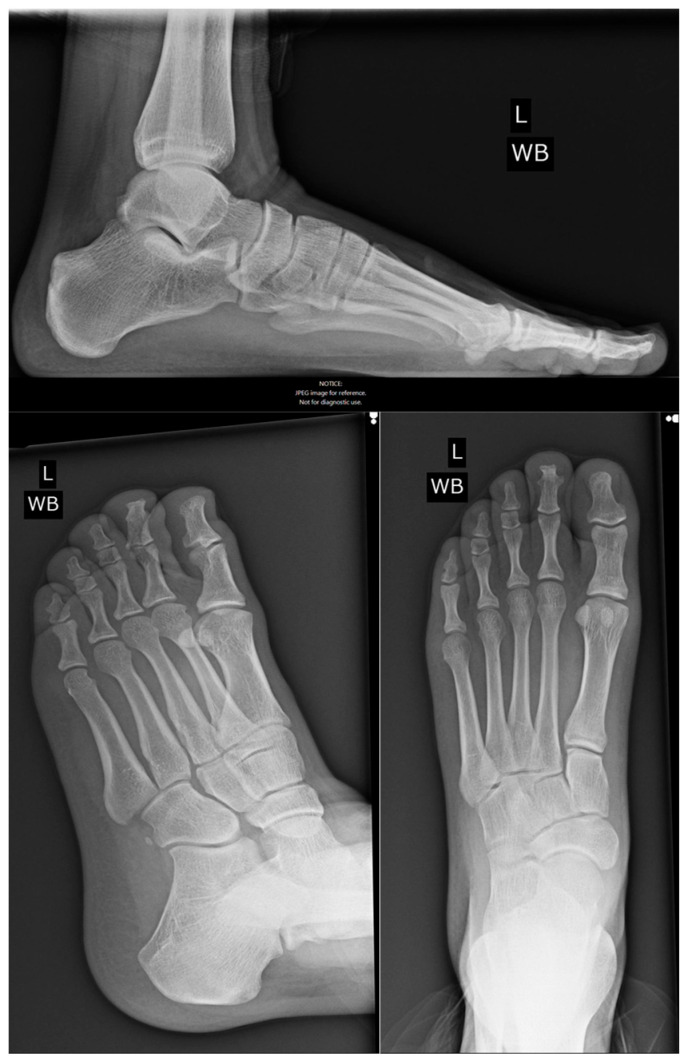
One-year postoperative radiograph showing maintained fusion and restored toe profile.

At his 1-year follow-up, radiographs confirmed maintained fusion, and clinical examination showed normalization of toe size with resolution of pain and restoration of athletic function (Figure 6 and Figure 7).

## 3. Discussion

CNO is an autoinflammatory bone disorder that frequently presents diagnostic uncertainty in pediatric patients. Clinical features such as localized pain, swelling, and functional limitation overlap substantially with infectious osteomyelitis, often resulting in prolonged or repeated courses of antibiotics despite negative microbiological cultures. The present case exemplifies this diagnostic challenge, as the patient underwent multiple antibiotic regimens over several years without sustained clinical improvement. Early biopsy demonstrated remodeling changes with scattered neutrophils, findings that are nonspecific and do not support active bacterial infection, yet the diagnosis of CNO was not initially considered.

Formal diagnostic criteria such as the Jansson or Bristol criteria were not explicitly applied in this case; rather, the diagnosis of chronic non-bacterial osteomyelitis was established by exclusion, based on the prolonged clinical course, repeatedly negative microbiological cultures, histopathologic findings consistent with sterile inflammation, characteristic imaging features, and lack of sustained response to prolonged antibiotic therapy. This diagnostic approach is consistent with current clinical practice, as no single laboratory, imaging, or histologic finding is pathognomonic for CNO. Diagnostic delay remains common, with prior studies reporting a median time to diagnosis of up to two years, frequently accompanied by unnecessary antibiotic exposure and repeated hospital evaluations [4,16]. The protracted interval between symptom onset and definitive diagnosis in this patient emphasizes the ongoing need for increased clinical awareness of CNO among pediatric and surgical providers.

Prior literature highlights the persistence of this issue. Oliver et al. reported that among 250 children with confirmed CNO, none demonstrated a meaningful response to antibiotics, and a considerable proportion received therapy for six months or longer [16]. Similar findings have led to increased awareness within the fields of rheumatology and orthopedics. Educational initiatives targeting primary care and surgical teams were associated with reduced diagnostic delays, as shown in a regional improvement project by Roderick et al. [4]. Improved access to advanced imaging, standardized diagnostic pathways, and early involvement in pediatric rheumatology may further reduce unnecessary antibiotic exposure and improve outcomes.

Although bisphosphonates and biologics are often effective for multifocal or systemic disease, localized osseous destruction of a small digit may require structural restoration to prevent deformity, joint instability, and potential amputation. In this patient, progressive resorption of the distal and middle phalanges indicated significant long-standing inflammation that had advanced beyond what medical management alone could address. Autologous calcaneal bone grafting with arthrodesis provided stable reconstruction of the toe and preserved limb function—an approach rarely reported for localized CNO but demonstrably successful here. Although bisphosphonates and biologics are often effective for multifocal disease, localized osseous destruction of a small digit may require structural restoration to prevent deformity, joint instability, and potential amputation. This case suggests that pediatric digits may respond favorably to surgical reconstruction even after prolonged disease duration. Given these considerations, CNO should be recognized promptly in pediatric patients with chronic, culture-negative osteomyelitis to avoid unnecessary antibiotic exposure and diagnostic delay. In addition, failure to appreciate progressive focal bone loss may postpone timely referral for surgical evaluation, whereas limb-sparing reconstruction may represent a viable option in selected cases in which localized osseous destruction compromises digital stability or function. Although few reports have described the surgical management of focal CNO in pediatric toes, additional research and longer-term follow-up are needed to establish indications, procedural outcomes, and the long-term durability of digital reconstruction.

Patient perspective: “My toe had been swollen for years, and I was told nothing could be performed. When the new X-ray showed that the bone was disappearing, I was very worried about possibly losing my toe. After the surgery, the pain went away, and my toe finally looks normal again. I’m able to run and play sports the way I used to. I’m grateful that the doctors looked deeper into the problem and offered a solution that saved my toe.”

## 4. Conclusions

CNO remains a challenging and often delayed diagnosis in pediatric patients with persistent or recurrent bone inflammation. This case illustrates how structural deterioration of a small bone, even in a single digit, can progress despite negative cultures and antibiotic therapy. Autologous calcaneal bone grafting with digital fusion provided a durable, limb-sparing solution that restored alignment, stability, and function of the affected limb. Early recognition of CNO in pediatric patients with culture-negative osteomyelitis may prevent prolonged diagnostic uncertainty and unnecessary antibiotic exposure. Although most cases of CNO respond to medical therapy, this report demonstrates that surgical reconstruction can serve as a viable limb-salvage strategy in selected patients with progressive focal osseous loss. Larger case series and long-term studies are needed to define surgical indications, evaluate complication rates, and determine the durability of such reconstructions in pediatric populations.

## Data Availability

The original contributions presented in this study are included in the article. Further inquiries can be directed to the corresponding author.

## Abbreviations

The following abbreviations are used in this manuscript:

CNO	Chronic non-bacterial osteomyelitis
CRMO	Chronic recurrent multifocal osteomyelitis
MRI	Magnetic resonance imaging
NSAID	Nonsteroidal anti-inflammatory drug
DMARD	Disease-modifying antirheumatic drugs
ED	Emergency department
ESR	Erythrocyte sedimentation rate
CRP	C-reactive protein
